# Fructo-Oligosaccharide (DFA III) Feed Supplementation for Mitigation of Mycotoxin Exposure in Cattle—Clinical Evaluation by a Urinary Zearalenone Monitoring System

**DOI:** 10.3390/toxins10060223

**Published:** 2018-06-01

**Authors:** Katsuki Toda, Seiichi Uno, Emiko Kokushi, Ayaka Shiiba, Hiroshi Hasunuma, Daisaku Matsumoto, Masayuki Ohtani, Osamu Yamato, Urara Shinya, Missaka Wijayagunawardane, Johanna Fink-Gremmels, Masayasu Taniguchi, Mitsuhiro Takagi

**Affiliations:** 1 United Graduate School of Veterinary Sciences, Yamaguchi University, Yamaguchi 753-8515, Japan; t1643eson@yahoo.co.jp (K.T.); shiibaayakahime@yahoo.co.jp (A.S.); masa0810@yamaguchi-u.ac.jp (M.T.); 2 Shepherd Central Livestock Clinic, Kagoshima 899-1611, Japan; hasu@fa3.so-net.ne.jp (H.H.); papashepherd@gmail.com (D.M.); 3 Faculty of Fisheries, Kagoshima University, Kagoshima 890-0056, Japan; uno@fish.kagoshima-u.ac.jp (S.U.); kokushi@fish.kagoshima-u.ac.jp (E.K.); 4 Nippon Beet Sugar Manufacturing Co., Ltd., Obihiro 080-0835, Japan; mohtani@nitten.co.jp; 5 Joint Faculty of Veterinary Medicine, Kagoshima University, Kagoshima 890-0062, Japan; osam@vet.kagoshima-u.ac.jp; 6 Soo Agricultural Mutual Aid Association, Kagoshima 890-8212, Japan; urara@nosai-soo.com; 7 Department of Animal Science, University of Peradeniya, Peradeniya 20400, Sri Lanka; missakaw@pdn.ac.lk; 8 Faculty of Veterinary Medicine, Utrecht University, Yalelaan 104, The Netherlands; J.Fink@uu.nl; 9 Laboratory of Theriogenology, Joint Faculty of Veterinary Medicine, Yamaguchi University, Yamaguchi 753-8515, Japan

**Keywords:** cattle, DFA III, mycotoxin, tight-junction, urine, blood

## Abstract

The potential effect of difructose anhydride III (DFA III) supplementation in cattle feed was evaluated using a previously developed urinary-zearalenone (ZEN) monitoring system. Japanese Black cattle from two beef herds aged 9–10 months were used. DFA III was supplemented for two weeks. ZEN concentrations in feed were similar in both herds (0.27 and 0.22 mg/kg in roughage and concentrates, respectively), and below the maximum allowance in Japan. ZEN, α-zearalenol (α-ZOL), and β-ZOL concentrations in urine were measured using LC/MS/MS the day before DFA III administration, 9 and 14 days thereafter, and 9 days after supplementation ceased. Significant differences in ZEN, α-ZOL, β-ZOL, and total ZEN were recorded on different sampling dates. The concentration of inorganic phosphate in DFA III-supplemented animals was significantly higher than in controls on day 23 (8.4 vs. 7.7 mg/dL), suggesting a possible role of DFA III in tight junction of intestinal epithelial cells. This is the first evidence that DFA III reduces mycotoxin levels reaching the systemic circulation and excreted in urine. This preventive effect may involve an improved tight-junction-dependent intestinal barrier function. Additionally, our practical approach confirmed that monitoring of urinary mycotoxin is useful for evaluating the effects of dietary supplements to prevent mycotoxin adsorption.

## 1. Introduction

Contamination of agricultural commodities with mycotoxins, which are secondary fungal metabolites, is a major worldwide problem in agriculture and livestock production [1]. Consumption of mycotoxin-contaminated products is generally believed to cause acute and chronic effects in humans and animals; thus, contamination of food, feed, and ingredients by mycotoxins poses significant health risks [1,2]. Recently, we found mycotoxin contamination in the dietary feed (rice straw) of a cattle herd whose urine was shown to contain zearalenone (ZEN) produced by *Fusarium* spp., and its secondary metabolites, by means of our liquid chromatography-tandem mass spectrometry (LC-MS/MS) monitoring technique [3]. Moreover, we suggested that monitoring ZEN levels in urine is a practical and useful way of evaluating the contamination status of a cattle herd and assessing the efficiency of the mycotoxin adsorbents (MAs) supplemented in dietary feed to impair intestinal adsorption of mycotoxins [3,4].

Several approaches for protecting animals from the toxic effects of natural mycotoxin contamination from both pre- and post-harvest products have been reported, such as appropriate field management and crop husbandry, the introduction of a non-toxigenic antagonistic fungal strain in the field prior to harvest, adequate storage management, and application of fungicidal agents and other protective silage additives at the post-harvest stage. In animal feed, the application of adsorbing agents, pro- and prebiotics, particularly yeast and yeast cell fractions, or mycotoxin-degrading enzymes has become common practice [5,6,7,8]. MAs generally consist of a mixture containing a mineral clay carrier, yeast cell wall preparations, and, in some cases, enzymes or living microorganisms (probiotics) that may adsorb and detoxify mycotoxins. We recently reported on the use of MAs and indicated their significant effects on the reduction in urinary ZEN concentration, concomitant with the reduction in the somatic cell count in a dairy cattle herd [4,9].

Recently, there has been a growing interest in the health-promoting benefits of prebiotics and non-digestible oligosaccharides, such as mannan-oligosaccharides [10,11], fructo-oligosaccharides (FOS) [12], and lactulose [13], to reduce the incidence of diseases in animals [14]. Difructose anhydride III (DFA III) is a naturally occurring, non-digestible disaccharide present in commercial roasted chicory that is manufactured from inulin by microbial fermentation [15,16]. DFA III promotes calcium absorption in rats [17,18], humans [19,20], and cattle [21,22]. Furthermore, Minamida et al. [23,24] reported that oral administration of DFA III in laboratory animals may help to maintain a healthy balance of intestinal microbiota; they suggested that DFA III is a novel candidate prebiotic. Additionally, we reported on the efficacy of DFA III supplementation as a prebiotic for the improvement in the health and intestinal microbiota of calves [25,26]. Recently, direct interactions of these oligosaccharides with intestinal epithelial cells have been reported, which have indicated that these oligosaccharides improve and protect the intestinal barrier integrity and modulate the immune responses of epithelial cells [27,28,29]. Based on these findings regarding the possible efficacy of oligosaccharides as prebiotics, we hypothesized that the etiotropic effects of DFA III could be applied to cattle herds in practice, and that the administration of DFA III would help to maintain good health, and improve and protect the intestinal barrier integrity in cattle as an alternative way to protect against the chronic low-dose mycotoxin contamination of cattle diets.

Therefore, the objectives of this field study were to evaluate the beneficial effects of supplementary DFA III on the intestinal barrier function in cattle, by monitoring urinary concentrations of ZEN and its metabolites as an indicator of the impact of DFA III on mycotoxin absorption. The concentrations of calcium (Ca), inorganic phosphate (Pi), and magnesium (Mg) were also measured in serum as indicators of optimal functioning of the intestinal epithelial cells.

## 2. Results

No significant clinical differences were observed between both cattle herds in this experiment.

### 2.1. Concentration of ZEN and Its Metabolites with or without Supplementation of DFA III

**Figure 1:**
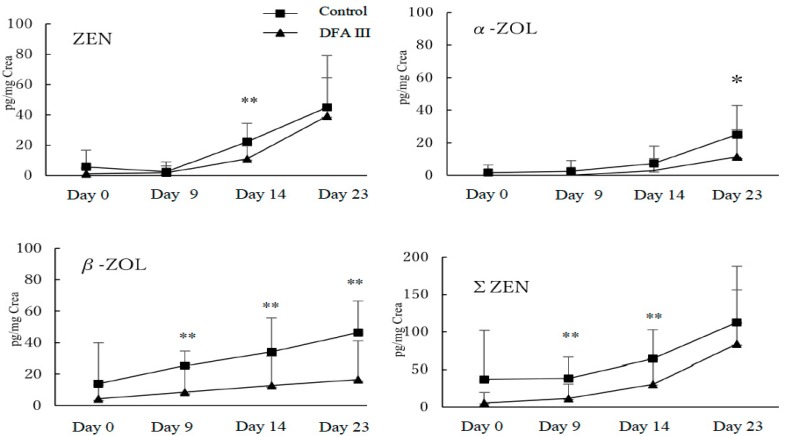
Results for the analysis of urinary concentrations of ZEN, α-ZOL, β-ZOL, and total ZEN (ΣZEN; ZEN + α-ZOL + β-ZOL) during the experimental period, with (DFA III group) or without DFA III supplementation (control group). * Significant difference (*p* < 0.05) between DFA III group and control group on each day. ** Significant difference (*p* < 0.01) between DFA III group and control group on each day.

Results for the analysis of urinary concentrations of ZEN, α-ZOL, β-ZOL, and total ZEN (ΣZEN; ZEN + α-ZOL + β-ZOL) during the experimental period, with and without DFA III supplementation, are shown in Figure 1.

Urinary ZEN concentrations on day 0 revealed that each herd had approximately the same level of contamination with ZEN. However, when comparing the DFA III-treated and control groups, significant differences (*p* < 0.05) were confirmed for ZEN on day 14 (11.0 ± 8.5 vs. 22.2 ± 12.2 pg/mg of Creatinine); for α-ZOL on day 23 (11.3 ± 16.7 vs. 25.2 ± 17.9 pg/mg of Creatinine), for β-ZOL on day 9 (8.6 ± 18.5 vs. 25.4 ± 9.3 pg/mg of Creatinine), on day 14 (12.7 ± 18.9 vs. 34.3 ± 21.5 pg/mg of Creatinine) and day 23 (16.5 ± 24.8 vs. 46.5 ± 19.9 pg/mg of Creatinine); and for ΣZEN (ZEN + α-ZOL + β-ZOL) on day 9 (11.6 ± 19.5 vs. 38.3 ± 28.0 pg/mg of Creatinine) and day 14 (31.0 ± 33.3 vs. 63.9 ± 38.9 pg/mg of Creatinine).

### 2.2. Ca, Mg, and Pi Concentrations with or without Supplementation of DFA III

**Figure 2:**
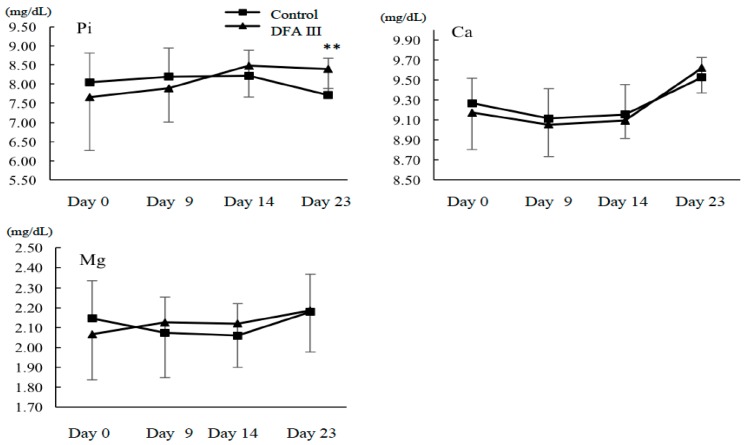
Results for the concentration of serum Pi, Ca, and Mg during the experimental period with (DFA III group) and without DFA III supplementation (control group). ** Significant difference (*p* < 0.05) between DFA III group and control group.

The results for the concentration of serum Ca, Mg, and Pi during the experimental period are shown in Figure 2. Although no differences were observed in either the serum Ca or Mg, the Pi concentration in DFA III-supplemented animals was significantly higher than that in the control animals on day 23 (8.4 ± 0.5 vs. 7.7 ± 1.0 mg/dL), suggesting a possible role of DFA III in the tight-junction functions of intestinal epithelial cells.

## 3. Discussion

Recently, Akbari et al. [27] reported that galacto-oligosaccharides (GOS) can prevent the typical adverse effects of the mycotoxin deoxynivalenol in a concentration-dependent manner. The effects of GOS, which facilitated tight-junction assembly and regulated claudine-3 gene expression, were of special interest and the authors further examined the microbiota-independent effects of oligosaccharides on intestinal epithelial cells and compared the effects of GOS and FOS. They reported that not only GOS, but also FOS showed a protective effect on deoxynivalenol-induced impairment of Caco-2 monolayer integrity, and they accelerated tight junction reassembly [29]. Based on these results and our previous reports regarding the clinical effects of DFA III [25,26], we hypothesized that DFA III has similar protective effects on the intestinal barrier functions and intestinal ion transport. We selected ZEN as a model compound/substance, again based on our previous results of mycotoxin exposure monitoring in cattle herds. The results reported herein indicated that, when comparing the DFA III and control groups, DFA III supplementation of dietary feed altered ZEN adsorption and, hence, excretion levels in cattle. Moreover, although no differences were observed in either serum Ca or Mg, the Pi concentration was significantly higher in the DFA III-supplemented animals than in the controls on day 23 (8.4 vs. 7.7 mg/dL), suggesting a role of DFA III in the tight junctions of intestinal epithelial cells.

Compared with the results of our previous report [9], in the present study, there was a much clearer reduction in β-ZOL than in ZEN or α-ZOL in the DFA III group compared with that in the control group, and there were different ratios of ZEN and its metabolites. Additionally, significant differences among ZEN, α-ZOL, and β-ZOL at the different sampling times were obtained. Malekinejad et al. [30] reported differences between species in the hepatic biotransformation of ZEN and demonstrated that β-ZOL is the dominant hepatic metabolite in cattle. Therefore, our results may also reflect the alteration in ZEN (parent) adsorption in the DFA III group compared with that in the control group. Regarding the urinary concentrations of ZEN and its metabolites (α-ZOL and β-ZOL), and the differences obtained among the sampling times, although the reason for the differences between reports is obscure, we assume that age differences, variations in ZEN contamination levels, or both may have affected liver metabolism. Further research taking into account cattle age and ZEN contamination levels are warranted.

Strategies for the detoxification of mycotoxin-contaminated feed in a cost-effective way are still poorly developed, and the most promising approach for reduction in the risk of mycotoxin exposure remains the use of non-nutritive adsorptive materials in animal diets [31]. It was previously stated that ruminating animals develop mycotoxicoses at low frequencies, as the rumen flora act as a first line of defense against mycotoxins at the usual levels of exposure in cattle herds. However, drastic changes in feed composition and a high percentage of protein in dairy diets have modified the detoxification capacity of rumen microorganisms. Therefore, MA supplementation is a widely used approach to reduce the risk of mycotoxicosis even in cattle [1,9]. Moreover, accumulating evidence in the last decade suggests that one of the major organs suffering the adverse effects of mycotoxins is the gastrointestinal tract, and more specifically the intestinal barrier. Disturbance of intestinal barrier integrity results in the translocation of feed antigens and even pathogens into the surrounding tissue, causing an intense and often systemic inflammatory response [32,33]. It has been suggested that glutamine, L-arginine, various fatty acids, and particularly non-digestible oligosaccharides play a role in the regulation of intestinal barrier function, and may have potential applications for the prevention and treatment of diseases associated with intestinal barrier impairment [34,35,36]. We have previously demonstrated the usefulness of urine analysis for the objective evaluation of the effects of supplemental feed additives on the bioavailability of mycotoxins [9].

In the present study, our in vivo results clearly indicated that DFA III (part of the FOS family) showed a capacity to protect intestinal epithelial cells against ZEN in cattle. In addition, the significantly higher Pi concentration in DFA III-supplemented animals than in control animals, after the DFA III supplemental period, suggests a possible role of DFA III in the tight-junction functions of intestinal epithelial cells. DFA III is an indigestible oligosaccharide that is enzymatically synthesized from inulin [37]. In vitro experiments conducted on the small intestines of rats [17,38] and duodena of cows [22] have shown that Ca absorption via the paracellular pathway can be accelerated by agents that act upon tight junctions. DFA III promotes paracellular transport by reducing transepithelial electrical resistance (TEER) and enhancing the transport of paracellular markers [39,40], with alterations to claudin-1, a component of tight junctions and actin filaments in Caco-2 cells [40]. The tight junctions play a crucial role in paracellular nutrient transport, as well as barrier function in the intestines. The paracellular route largely contributes to the transport and absorption of certain minerals, such as calcium and magnesium [34,41]. The modification of tight-junction structure and function by DFA III may influence the paracellular absorption of these essential elements. Meanwhile, these minerals are also known to be absorbed by the active transcellular pathway in the intestine. The contribution of each pathway depends on the dietary level of the minerals, and transcellular transport is generally more tightly regulated than in paracellular transport [34]. Therefore, transcellular transport may compensate for changes in the paracellular transport of ions. Further study is required to clarify the effects of DFA III in cattle, and some of the research priorities include the investigation of optimal DFA III supplemental volume and method of application in cattle herds. 

## 4. Conclusions

In conclusion, supplementation of dietary feed with DFA III altered ZEN and differentially affected ion adsorption levels in cattle. Our results are the first clear indication that DFA III supplementation can reduce the levels of mycotoxins that reach the systemic circulation and are excreted in the urine. This preventive effect may be associated with an improved tight-junction-dependent intestinal barrier function. Furthermore, our practical approach confirmed that monitoring urinary mycotoxin is useful for the evaluation of the effects of dietary supplements that may prevent mycotoxin adsorption. Further field studies are in progress to create a database for the assessment of DFA III as a dietary supplement to reduce mycotoxin absorption in cattle and other animal species.

## 5. Materials and Methods

The experiments were conducted according to the regulations concerning the protection of experimental animals and the guidelines of Yamaguchi University, Japan (No.40, 1995, approval date 27 March 2017). 

### 5.1. Chemicals and Solvents

DFA III was kindly donated from Nippon Beet Sugar Manufacturing Co. Ltd., Obihiro, Japan. ZEN was purchased from MP Biomedicals (Heidelberg, Germany). The metabolites α-ZOL and β-ZOL were purchased from Sigma (St. Louis, MO, USA). Stock solutions of ZEN, α-ZOL, and β-ZOL, each at a concentration of 1 μg/mL in methanol, were stored under light protection at 4 °C. High performance liquid chromatography (HPLC)-grade methanol was purchased from Wako Pure Chemicals Industries, Ltd. (Osaka, Japan). β-Glucuronidase/arylsulfatase solution was purchased from Merck (Darmstadt, Germany). Sodium acetate was purchased from Kanto Chemical Co. Inc. (Tokyo, Japan) and Tris was purchased from Nakalai Tesque Inc. (Kyoto, Japan). 

### 5.2. Japanese Black Cattle Herds and Sample Collection

Japanese Black heifers from two beef herds (Herd 1: *n* = 10, Herd 2: *n* = 20, 10 months old, 250−300 kg) raised in Kagoshima Prefecture, Japan, were included in this experiment. Herds 1 and 2 consisted of 370 and 500 beef cattle, respectively. Basically, Herds 1 and 2 were fed with purchased concentrate and rice straw. The detailed compositions of the dietary feed of the two herds are shown in Table 1.

In both herds, the roughages and concentrates were stored at ambient temperature, in feed sheds and silos, respectively. The ZEN level in the dietary feed of both herds was provisionally measured before the beginning of the experiment, via LC-MS/MS, as previously reported [9]. The concentration of ZEN in the mixture of roughages and concentrates fed to heifers was 0.27 mg/kg in Herd 1 and 0.22 mg/kg in Herd 2. These findings confirmed that the contamination of dietary feed with ZEN was below the threshold levels allowed by Japanese regulations (<1.0 ppm), and were very similar in both herds.

Two groups of heifers were randomly selected from each experimental Herd and divided into two treatments that differed in feed supplementation as follows: DFA III group (Herd 1: *n* = 5, Herd 2: *n* = 10) was fed 40 g DFA III/day (20 g each feeding time) mixed with concentrate, and the control group (Herd 1: *n* = 5, Herd 2: *n* = 10) was fed with no DFA III supplementation. This dose of DFA III is the recommended dose for the prevention of hypocalcemia in dairy cows [21,22], which may affect tight-junction functions.

**Figure 3:**
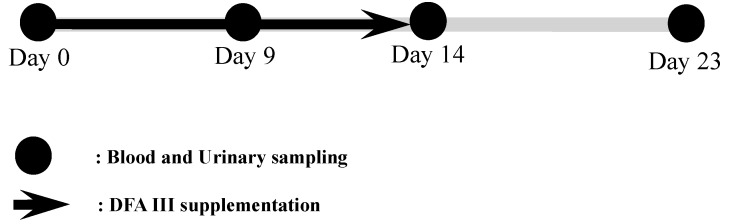
Experimental protocol for DFA III supplementation in dietary feed and sampling of blood and urine.

Two hours after the morning feeding, urine samples were collected from the animals by massaging the pudendum, and blood samples were collected from the jugular vein in silicone-coated tubes. This sampling was performed at the start of DFA III supplementation (Day 0), 9 days (day 9), and 14 days (day 14, i.e., on the last day of DFA III supplementation) after treatment initiation, and on the last day of the experimental period, 23 days (day 23) after treatment initiation. In addition, samples of rice straw and feed concentrate (approximately 1 kg each) were obtained from both herds to measure ZEN concentration in the feed. The protocol for DFA III supplementation and all sampling procedures is summarized in Figure 3. 

All samples were immediately placed in a cooler containing dry-ice for protection from light and transported to the laboratory. The urine and blood samples were centrifuged at 1000× *g* and 2000× *g,* respectively, for 10 min at room temperature. The urine and serum samples were frozen at −30 °C until the analysis of ZEN and its metabolites, urine creatinine (as a reference for the correction of urine volume), and the serum ion concentrations of Ca, Mg, and Pi, using a Labospect 7180 autoanalyzer (Hitachi, Japan).

### 5.3. Methods of Urine Sample Analysis

All urine samples were analyzed by LC/MS/MS, as described in our previous report [4]. Briefly, 0.5 mL of each urine sample was mixed with 3.0 mL of 50 mM ammonium acetate buffer (pH 4.8) and 8 μL of glucuronidase/arylsulfatase solution, and incubated for 12 h at 37 °C. The solution was loaded onto a C18 SPE column, which was preconditioned with 3 mL 100% methanol and 2 mL of Tris buffer, followed by the addition of 2 mL Tris buffer and 3 mL of 40% methanol. After washing the SPE column with approximately 1 mL of 80% methanol, the volume of the eluted solution was adjusted to exactly 1 mL. Then, 20 μL of the reconstituted solution was injected into the LC/MS/MS system. The LC/MS/MS analyses were performed on an API 2000 LC/MS/MS system (Applied Biosystems, Foster City, CA, USA) equipped with an electrospray ionization interface and a 1200 Infinity Series HPLC system (Agilent Technologies, Santa Clara, CA, USA). The detection limits for ZEN, α-ZOL, and β-ZOL were 0.04 ng/mL, 0.05 ng/mL, and 0.05 ng/mL, respectively, while the mean recovery rates for ZEN, α-ZOL, and β-ZOL were 90%, 109%, and 90%, respectively. The urine creatinine concentrations were determined by using a commercial kit (Sikarikit-S CRE, Kanto Chemical, Tokyo, Japan), according to instructions by the manufacturer, and were measured using the 7700 Clinical Analyzer (Hitachi High-Tech, Tokyo, Japan). All urine concentrations were expressed as a ratio of creatinine (pg/mg creatinine), as described previously [4]. 

### 5.4. Statistical analysis

The results for ZEN, α-ZOL, β-ZOL, and ΣZEN (ZEN + α-ZOL + β-ZOL) concentrations and serum ion concentrations are expressed as means ± SD. The urine and serum values of the DFA III and control groups were compared using Student’s t-tests with Welch’s correction when the variances differed. *P* values less than 0.05 were considered to indicate a statistically significant difference.

## Figures and Tables

**Table 1:** Composition of feed provided to the two herds kept for fattening purposes.

Herd	Forage Feed, kg	Formula Feed						
		Total, kg	Bran, %	Cereal, %	Oil Seed Meal, %	Other, %	TDN, %	CP, %
Herd 1	Straw 2.0, Timothy grass 2.0	3	24	46	16	14	>70.0	>16
Herd 2	Straw 2.0, Oats 2.0	2	27	56	7	10	>71.5	>14

TDN: total digestible nutrients, CP: crude protein.
